# COVID-19: The Dentists’ Perceived Impact on the Dental Practice

**DOI:** 10.1055/s-0040-1721910

**Published:** 2021-02-23

**Authors:** Alessandra Amato, Carolina Ciacci, Stefano Martina, Mario Caggiano, Massimo Amato

**Affiliations:** 1 Department of Medicine, Surgery and Dentistry, Scuola Medica Salernitana, Baronissi, Salerno, Italy

**Keywords:** COVID, dental practice, dentistry, survey

## Abstract

**Objectives** The present study aimed to investigate the dentists’ knowledge of the risks from the severe acute respiratory syndrome coronavirus 2 (SARS-CoV-2) infection, and how it will impact their practice.

**Materials and Methods** An ad hoc self-administered anonymous questionnaire was submitted to Italian dentists.

**Statistical Analysis** Differences in rates were calculated using the chi-square test. The level of significance was set at *p* < 0.05.

**Results** A total of 849 dentists fully completed the survey. Eighty-eight per cent of Italian dentists are worried about the health of their families, with no difference in high-risk (red zone) and low-risk (orange zone) regions. About 86% of professionals report some income loss and 94% fear a drop in patients after the quarantine phase, with the dentists working the red zone claiming a greater economic loss and fearing a reduced practice after the end of the lockdown.

**Discussion** A large majority of the Italian dentists appears to be aware of the need for changes in their dental practices by planning specific sterilization processes between appointments, testing patients for SARS-CoV-2 serology, asking patients not to be accompanied, and reducing the number of visits per day.

**Conclusion** The survey expresses the serious concern of the dentists for the pandemic’s effects on their profession.

## Introduction

A new viral infection outbreak first occurred in China in December 2019 and rapidly became a global health emergency. 1 On January 9, 2020, the new severe acute respiratory syndrome coronavirus 2 (SARS-CoV-2)/human/Wuhan/X1/2019 was identified as the pathogen responsible for the pandemic declared by the World Health Organization (WHO) on March 11, 2020. 2

SARS-CoV-2 causes COVID-19, an acronym that derives from the synthesis of the terms coronavirus disease and the year of its identification 2019. 3

The most frequently encountered symptoms of COVID-19 are asthenia, myalgias, rhinitis, pharyngitis, dry cough, dyspnea, worsening fever, and diarrhea.

However, it is now clear that SARS-CoV-2 has infected a sensitive number of people presenting little or no symptoms at all, especially those of a young age. The contagion occurs mainly through the respiratory droplets when infected people in close contact sneeze or cough. 4 Although the virus is more contagious when the patient is symptomatic, the transmission is possible from human to human even in patients with mild and/or without symptoms. 5

In the COVID-19 era, the WHO and all governments recommend robust containment and control activities to reduce the spread of this virus. The WHO, national agencies, and in Italy, the National Institute of Health regularly update the guidelines. One of the recommendations is the use of e-health technology to reduce viral transmission, which can quickly occur in traditional health care settings. Previous studies have described the use of “telemedicine,” also referred to as “telehealth,” in several clinical settings to facilitate remote health care. 6 7

In most countries, telehealth has acquired great importance in the COVID-19 outbreak by helping patients and doctors reduce as much as possible the traditional routine outpatient visits, warranting social distancing also in hospitals by admitting only the most severe cases. Indeed, during quarantine, most of the dentists attended only urgency/emergency treatments. 8

Telehealth and remote consultations are useless in a dental practice. In the dentistry setting, the viral spread is unquestionable due to the proximity between a patient’s mouth and the operator (a working distance of ~30 cm). 9 Moreover, the constant atomization of the air and water necessary for cooling and removing the residues of dental tissue or materials, obtained from commonly used dental instruments, may contribute to the spread of potentially infected droplets within an arc of ~3 m from the work area. 10

Therefore, a change in dental practice will be necessary by following the developing guidelines to help dentists minimize the risks of the SARS-CoV-2 infection.

The present study aimed to investigate the dentists’ knowledge of the risks from the SARS-CoV-2 infection, and how it will impact their practice.

## Materials and Methods

### Investigation Questionnaire

We developed an ad hoc self-administered anonymous questionnaire for the dentists. We forwarded the survey throughout Italy via Internet from April 26 to May 3, 2020. The homepage of the survey provided information on the scope and purpose of the study. The dentists read and agreed to the utilization of the data provided.

As communication tools, we used professional WhatsApp or e-mail lists to evaluate the sample size of the respondents/invitees. There were no incentives for participation.

The questionnaire consisted of 25 tems, which assessed the following: demographic information, management information, any changes or solutions for the resumption of professional activity, and possible investments to work safely for both the operator and the patient. Attitudes toward the COVID-19 infection were studied using an ordinary scale. In particular, we asked if the dentists were concerned about themselves, their family members, and their team regarding the COVID-19 infection, if they had come in contact with the pathogen, and therefore, if they had symptoms and if they had gone to the emergency room for the alleged contagion.

Participants also had the opportunity to add additional comments at the end of the questionnaire. We calculated the average time of 5 minutes to complete it.

### Statistical Analysis

The categorical variables were expressed as frequencies. Differences in rates were calculated using the chi-square test. The significance level was set at *p* < 0.05. All the analyses were performed with commercial software (SPSS version 22.0; SPSS IBM, Armonk, New York, United States).

## Results

The lockdown started in Italy on February 23 in the northern regions and on March 11, it was extended to the entire country and the Italian government declared COVID-19 emergency. Therefore, 2,480 dentists received the survey after about 4 weeks of the lockdown. A total of 851 answered, but 849 fully completed the questionnaire. Among these 261 respondents were women, 588 were men, and there were 5 unspecified genders. Due to the small number of unspecified gender respondents, we distributed them among the men and women (three to men and two to women, based on the ratio of male/female respondents). According to the region of practice, we divided the participants into two groups:

- Red, if they lived in the so-called red zones of northern Italy, where the frequency of the COVID-19 was high.
- Orange, if they lived in the orange zones, the ones in the center and south of Italy. In the orange zones, the lockdown measures (smart working at home, the closing of schools and shops) reduced the virus spread. Therefore, the frequency of COVID-19 and its fatal outcomes were more than 10 times lower than in the red zones.

The demographics and characteristics of the responders are summarized in Table 1 .

**Table 1:** 

	All *N* = 849 (100%)	Women *N* = 261 (100%)	Men *N* = 588 (100%)	*p* -Value
Age groups, y				
25–29	14.8%	25.7%	10%	**0.0001**
30–49	39.1%	49.4%	34.5%	
>50	46.1%	24.9%	55.4%	
Living in red zones	34.0%	37.5%	32.5%	0.088
Living in orange zones	66%	62.5%	67.5%	
Note: Statistically significant differences are indicated in bold type.				

Table 2 shows the personal, family, and professional concerns about COVID-19, divided by region of practice. About 10% of the total number of professionals underwent testing (swab or serum) for COVID-19, mostly in the red zone. A greater proportion of dentists from the orange zone compared with those in the red were worried about the safety of their collaborators.

**Table 2:** Personal, family, and professional concerns about COVID-19.

	Total number *N* = 849	Red zone *N* = 289	Orange zone *N* = 560	*p* -Value
Number of those who in the last month visited the doctor because of COVID-19 symptoms	3.1%	5.5%	1.8%	**0.003**
Number of those who suffered from respiratory symptoms in the last month	5.8%	10%	3.6%	**0.0001**
Number of those admitted to the hospital because of respiratory symptoms	0.5%	1.4%	0	0.013
Number of those tested for SARS-CoV-2 (nasal swab or plasma IgG/IgM)	9.8%	15.9%	6.6%	**0.0001**
Number of those worried for the health of the family because of the COVID-19 concerns	88.3%	73.4%	80.9%	**0.008**
Number of those worried for the health of the collaborators because of the COVID-19 concerns	73.9%	68.5%	76.6%	**0.007**
Abbreviations: COVID-19, coronavirus disease 2019; Ig, immunoglobulin; SARS-CoV-2, severe acute respiratory syndrome coronavirus 2. Note: Statistically significant differences are indicated in bold type.				

Table 3 shows the substantial numerical difference between dentists who carry out their profession in public or private facilities (6.5 vs. 93.5%). The datum aligns with those of census, “Censis-RBM Health Insurance Report” on public, private, and intermediated health care in Italy (Welfare Day June 6, 2018) with 95% of the care provided by private dental practices.

**Table 3:** Substantial numerical difference between dentists

	All *N* = 849 (100%)	Women *N* = 261 (100%)	Men *N* = 588 (100%)	*p* -Value
**Working in**				
Public hospitals	7.2%	8.8%	6.5%	0.141
Private practice	92.8%	91.2%	93.5%	
Claiming a pecuniary loss because of the COVID-19	86.5%	80.1%	89.3%	**0.0001**
Fearing a reduced practice after the end of lockdown	94.1%	94.3%	94%	0.523
Esteemed percentage of reduction				
20%	12.8%	14.9%	11.9%	0.235
40%	33.6%	34.9%	33.0%	
50%	35.8%	36%	35.7%	
75%	17.8%	14.2%	19.4%	
Dental chairs				
1	19.2%	19.9%	18.9%	0.589
2	44.3%	46.4%	43.4%	
3	21.9%	21.5%	22.1%	
>3	14.6%	12.3%	15.6%	
Note: Statistically significant differences are indicated in bold type.				

Our data indicate that there was a linear correlation between the number of dental units and the perception of the damage. The average type of dental practice of the respondents is equipped with between one and two dental units (62.3%). About 86% of professionals report some income loss and 94% fear a drop in their caseload after the quarantine phase. However, the number of dentists with a single work station that claim no perception of money loss (20%) is greater than that of the professionals with three or more dental units (14%). The difference is also likely due to the greater incidence of expenses relating to personnel and management.

Table 4 shows the perception of economic loss by the red or orange zone. The most relevant findings are that dentists practicing in the red zone claim a greater economic loss and fear a reduced practice after the end of the lockdown than those living in the orange zone. It has been noted that dentists living in the red zone claim a greater number of dental chairs in their offices than those living in the orange zone.

**Table 4:** The perception of economic loss by the red or orange zone

	All *N* = 849 (100%)	Red zone *N* = 289 (100%)	Orange zone *N* = 560 (100%)	*p* -Value
**Working in**				
Public hospitals	7.2%	7.3%	7.1%	0.524
Private practice	92.8%	97.7%	92.7%	
Claiming a pecuniary loss because of the COVID-19	86.5%	92%	83.6%	**0.0001**
Fearing a reduced practice after the end of lockdown	94.1%	92%	95.2%	**0.048**
Esteemed percentage of reduction				
20%	12.8%	18.3%	10%	**0.0001**
40%	33.6%	38.1%	31.3%	
50%	35.8%	32.5%	37.5%	
75%	17.8%	11.1%	21.3%	
Dental chairs				
1	19.2%	12.1%	22.9%	**0.0001**
2	44.3%	40.5%	46.3%	
3	21.9%	26.68%	19.5%	
>3	14.6%	20.8%	11.4%	
Note: Statistically significant differences are indicated in bold type.				

Table 5 shows the measures that dentists will implement when returning to their practices post-lockdown. Only 13.9% of participants planned to increase the number of employees, but 44.9% would be willing to do it in case of lower taxation. The vast majority of respondents declared that they would change their agenda, ask patients not to be accompanied, use a COVID-19-oriented anamnestic fact sheet, and plan specific sterilization and sanitization processes for surfaces and equipment between appointments. The majority of dentists plan to invest in equipment to purify the air and surfaces of the office, but only 36.7% plan to seek professional advice for the safety changes at the office.

**Table 5:** Future measures for dentists to implement post-lockdown

	Total number *N* = 849 (100%)	Red zone *N* = 289 (100%)	Orange zone *N* = 560 (100%)	*p* -Value
Planning to increase the number of employees to face the emergency	13.9%	10%	15.9%	**0.012**
Willing to increase the number of employees to face the emergency only in case of lower taxation	44.9%	37.7%	48.6%	**0.002**
Willing to change the whole agenda of the office	94.1%	94.8%	93.8%	0.324
Willing to ask the patient not to be accompanied for the procedures	97.3%	95.8%	98.0%	**0.054**
Willing to prepare an anamnestic fact sheet COVID-19 oriented	94.9%	94.8%	95%	0.512
Willing to exhibit and inform the patients of the preventive and safety devices present in the office	92.7%	93.1%	92.5%	0.438
Planning a dressing and undressing path for the operator and assistants	82.7%	79.6%	84.3%	**0.054**
Planning a dressing and undressing path for the patients	55.1%	58.5%	53.4%	0.090
Planning specific sterilization and sanitization process for surfaces and equipment between appointments	95.1%	96.2%	94.5%	0.176
Planning of investing in equipment to purify the air and surfaces of the office	62.9%	57.4%	65.7%	**0.01**
Planning to search for professional advice for the additional safety measures at the office	62.3%	62.3%	62.3%	0.525
Planning to test the patients for SARS-CoV-2 serology	81.9%	84.4%	80.5%	0.096
Abbreviation: SARS-CoV-2, severe acute respiratory syndrome coronavirus 2. Note: Statistically significant differences are indicated in bold type.				

The statistical analysis reveals that the perception of the economic loss is obviously greater in the private sector than in the public one ( *p* = 0.0001). The results of a logistic regression analysis, entering the values of sex, age, number of dental units, and perception of the financial loss, reveal that males claim a greater perception of loss than females (borderline significance), the older ones more than the young people, and those who have over three dental units more than the others, gender, *p* = 0.102 (95% confidence interval [CI]: 0.930–2.220); age, *p* = 0.0001 (95% CI: 1.542–2.735); number of dental chairs *p* = 0.005 (95% CI: 1.106–1.760).

## Discussion

The survey of this study, completed during the quarantine for the COVID-19 outbreak in Italy, reveals that dentists had a strong perception of the damage to their practice not only during the lockdown time but also for the postpandemic period. Previous studies demonstrated that the return to their activity was a source of anxiety for dentists during COVID-19 outbreak. 10 11 Another study performed in Italy revealed that dentists believed that dental procedures could represent a high risk of infection for the operators, mainly due to aerosol produced. 12 Most of the dentists favored implementation of protocols to reduce in-office COVID-19 contagion, such as efficient sanitizing procedures combined with the correct use of personal protective equipment and a careful screening of each patient before entering the dental office. 13

This study focuses on dentists’ expectations of the economic and organizational impact of COVID-19 on the dental practice.

Among respondents, men in the over 50 years age group replied more frequently than women of the same age. In contrast, the women who responded were mostly in the 25 to 49 years age group. The men claimed an economic/financial loss more frequently than the women. However, men were older and more frequently working in larger offices than women. However, the datum agrees with the previsions of feminization of the dental practice, showing the future image of the profession. 14 15 Moreover, the dentists living in the red zone and having a greater number of dental chairs in the office claimed economic loss because of the COVID-19 pandemic more than those living in the orange zone.

COVID-19 is a matter of concern, raising doubts about the time needed to eradicate the disease, the efficacy of the therapies, and the possibility of a vaccine. Most dentists claim to be willing to make immediate organizational changes in their work system by varying their work agenda and adopting and displaying new safety measures in their office. Almost all dentists, from both areas, are ready to ask patients to be unaccompanied to limit crowding in waiting rooms and respect social distancing. 16

In detail, many of the participants claim that they will prepare an anamnestic fact sheet for COVID-19. Slightly more than 80% think they will set up a changing area for operators. However, only 55% of all dentists have thought about a changeover zone for patients. The results could also be dependent on the size and type of studies that allow this opportunity.

Concerning further and specific sanitization and sterilization interventions on surfaces, equipment, and tools between business appointments, more than 95% of respondents consider it necessary and appropriate. While more than 65.7% of dentists living in the orange zone believe they will invest in equipment to purify the air and surfaces of the office, only ~57% of those in the red zone consider it essential, believing that usual caution is sufficient for standard clinical practice. More than 60% of respondents are willing to search for specialized consultants for the new sanitizing devices; the rest will trust their suppliers.

Less than one sixth of respondents would not hire new support staff in their dental offices, but 45% would increase the staff if there were a tax exemption for new employees. The finding suggests that there is a need for government support.

Finally, 80% of professionals want to perform the SARS-CoV-2 serological tests 17 on patients at their own expense.

The datum is of interest and should be considered by the new guidelines. The dental patient is often on a medium duration therapy. Therefore, planning the repetition of the test over time, in case of a possible new outbreak of viral activity, could represent an additional safety procedure.

## Conclusion

The survey expresses the serious concern of dentists for the pandemic’s effects on the profession. In our opinion, this traces a novel clinical recommendation for the serological test, when its sensitivity and specificity will be better defined.
